# Influence of the Valsalva maneuver on cardiac hemodynamics and right to left shunt in patients with patent foramen ovale

**DOI:** 10.1038/srep44280

**Published:** 2017-03-07

**Authors:** Enfa Zhao, Yafei Zhang, Chunmiao Kang, Hua Niu, Jing Zhao, Lei Sun, Baomin Liu

**Affiliations:** 1 Department of Ultrasound, Second Affiliated Hospital of Xi’an Jiaotong University, Xi’an, Shaanxi, China; 2 Department of General Surgery, Second Affiliated Hospital of Xi’an Jiaotong University, Xi’an, Shaanxi, China

## Abstract

The purpose of this study was to investigate the influence of the Valsalva maneuver (VM) on cardiac hemodynamics in patients with patent foramen ovale (PFO). Sixty-five patients who were highly suspected to have PFO were included. The changes in E, A, E/A ratio of mitral valve blood flow, E, A, E/A ratio of tricuspid valve blood flow, left ventricular end-diastolic volume, area and right atrial area during the resting state and the strain phase of the Valsalva maneuver were observed by transthoracic echocardiography (TTE). Statistical analyses were performed using SPSS Version18.0. Compared to the resting state, mitral valve diastolic velocity E and A peaks at the strain phase of the Valsalva maneuver significantly decreased (*P* < 0.05), left ventricular end diastolic volume(LVEDV) and area(LVEDA) decreased significantly (*P* < 0.05), while E/A ratio of mitral valve, tricuspid valve systolic velocity E and A peaks and E/A ratio remained unchanged (*P* > 0.05). PFO hemodynamic changes mainly occurred in the left ventricle when the Valsalva maneuver was performed. The Valsalva maneuver increased pressure in the chest, then pulmonary venous return was impeded, which resulted in left ventricular limited filling, and E and A peaks decreased. The pressure of the left ventricle and atrium was lower than that of the right side, which resulted in right-to-left shunt (RLS) through PFO.

Patent foramen ovale (PFO) is believed to be a common finding in adults according to a study of 965 normal adult hearts in postmortem studies^1^, with a prevalence of 27.3% for all ages. A recent paper reported that up to one in four adults may show right-to-left shunting during their adult life^2^. In fetuses, the foramen ovale acts as a communication between the left atria and right atria, and plays an important role in blood circulation between maternally oxygenated blood and the systemic circulation^3^. After birth, functional PFO closure occurs immediately, followed by anatomical closure of the channel between the left atria and right atria within the first month^3^. However, there were still about 25% foramen ovale presenting failure to close, which resulted in persistent RLS through a one-way flap mechanism when the right atrial pressure (RAP) was higher than that of the left atrium. A patent foramen ovale (PFO), the most common cause of right-to-left shunt, is usually insignificant in hemodynamics. Large-diameter PFO may serve as a channel for passage of vasoactive substances, thrombus, fat, and air from the venous to arterial circulation. It is reported that PFO was associated with numerous disease processes including paradoxical embolism in cryptogenic stroke^4^, transient ischemic attack (TIA)^5^, latypnoea-orthodeoxia syndrome^6^, decompression sickness, migraine with aura and other venoarterial embolic phenomena^7^. Transcranial Doppler and transesophageal echocardiography (TEE) or transthoracic echocardiography (TTE) with the micro-bubbles test were commonly used methods to detect right-to-left shunt both at rest and during the Valsalva maneuver^8^,^9^. Since the original maneuver was first used by Valsalva in 1704, the VM has been widely used in clinical practice^10^. In recent years, c-TTE with the Valsalva maneuver significantly increased positive incidence of right-to-left shunt in the diagnosis of patent foramen ovale^9^,^11^. Even current studies^10^,^12^,^13^ reporting the Valsalva maneuver and right-to-left shunt, still have not reached a consensus about possible mechanism. Our study aimed to evaluate the influence of the Valsalva maneuver on the cardiac hemodynamics and right-to-left shunt in patients with patent foramen ovale and to explore the possible mechanism.

## Methods

### Patients

The study population consisted of 65 patients(mean age 38.29 ± 1.63 years, 58 were <55 years old; 28 male patients and 37 female patients) with high clinical suspicion of patent foramen ovale who suffered from migraine, vertigo, transient ischemic attack (TIA), limb weakness, blurred vision and unknown causes of cerebral ischemia. All patients were enrolled from April 2015 to November 2015 in Xi’an, Shaanxi Province, China. All patients gave written consent prior to the examination. The study protocol was performed with approval by the ethics committee of the Second Affiliated Hospital of Xi’an Jiaotong University and was performed in accordance with the CONSORT 2010 guidelines. Written informed consent was obtained from all subjects. Before TTE with the micro-bubbles test, all patients were instructed to perform the standard Valsalva maneuver. Patients who were unable to perform the maneuver during the test because of coordination impairment were excluded from our study. Routine ultrasound, Computed tomography (CT), and magnetic resonance imaging (MRI) were used to rule out cardiac, intracranial, or extracranial arterial disease and pulmonary arteriovenous malformation.

### TTE with the microbubbles test

**Figure 1:**
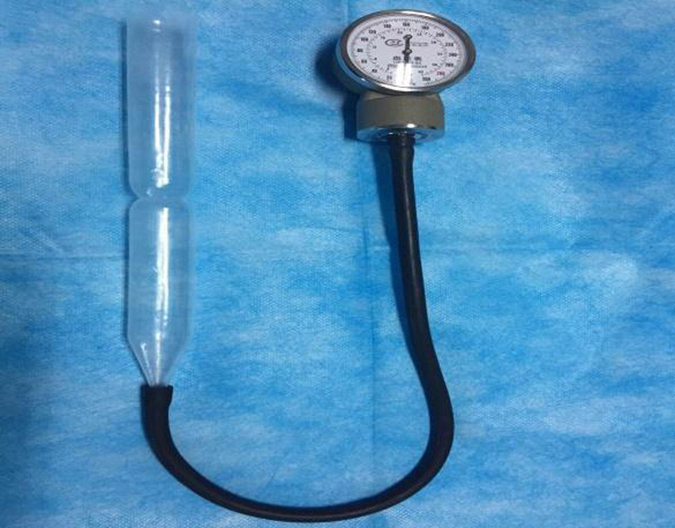
Simple pressure measurement device used in the study.

**Figure 2:**
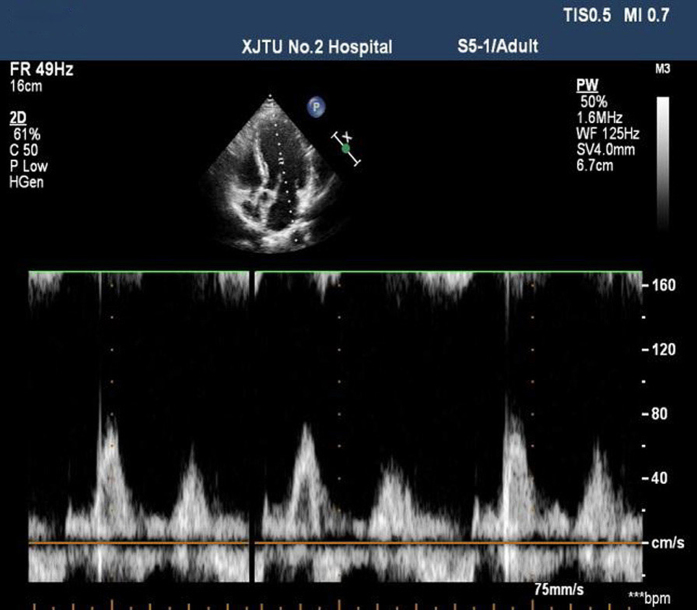
Measurement of E peak and A peak of mitral valve.

**Figure 3:**
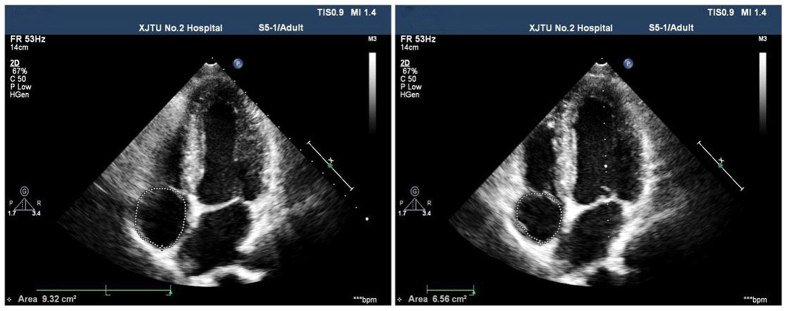
Measurement of the left ventricular end-diastolic volume and area using the modified Simpson’s rule in apical 4-chamber views at left ventricular end-diastole at rest and during the Valsalva maneuver.

**Figure 4:**
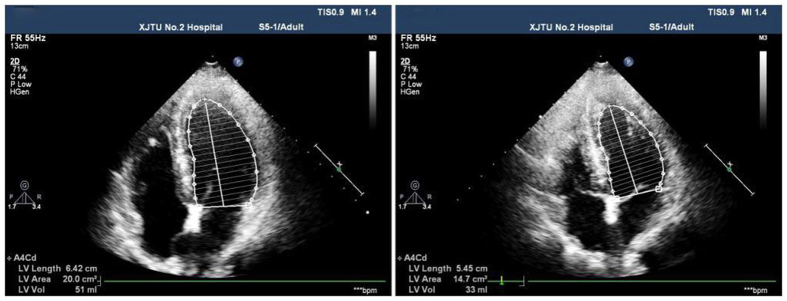
Measurement of the right atrial area in apical 4-chamber views at left ventricular end-systole at rest and during the Valsalva maneuver.

The simple pressure measurement device used in the study is shown in Fig. 1. It is consisted of a manometer and a small soft plastic tube connected by a rubber tube. Effective VM was evaluated by observing a minimum reading of 40 mmHg on the manometer^14^. The TTE with the micro-bubbles test was performed with Philips iE33 platform fitted with S5–1 transducer (1–5 MHz). The contrast agent was prepared with 1 mL air and 9 mL saline solution and was mixed between the two 10-mL syringes connected by a 3-way stopcock. Patients were told to remain in the left lateral position and instructed to breathe calmly. Routine echocardiography was performed to obtain the standard four-chamber view. Color Doppler sampling frame was placed at the tip of the mitral valve and tricuspid valve both at rest and during the Valsalva maneuver in order to get the blood flow spectrum of E and A peaks of mitral valve and E and A peaks of tricuspid valve. All parameters mentioned above were measured in three consecutive cardiac cycles to obtain the mean value, and E/A ratios were determined (Fig. 2). Recommendations for chamber quantification by the American Society of Echocardiography in 2005 were used to quantify the cardiac chamber size^15^. Two-dimensional measurements for area calculations using biplane method of disks (modified Simpson’s rule) in apical 4-chamber views at end diastole (LV EDD) at end systole (LV ESD), -at rest and during the Valsalva maneuver (Fig. 3). Right atrial areas were measured at left ventricular end-systole (Fig. 4). Patients were instructed to perform the standard Valsalva maneuver for maintaining a pressure of 40 mm Hg lasting 10–15 seconds, which was monitored by the pressure measurement mentioned above. As the lung inflated during the Valsalva maneuver and the ultrasound probe shifted accordingly, the apical 4-chamber view images moved or even disappeared. The image could not be track immediately. The probe should keep relatively fixed. When patients began to expire, the thoracic cage restored to the original position, and the apical 4-chamber view images could redisplay. This process usually took 2–3 cardiac cycles. Patients were required to rest for 5 minutes, and then the Valsalva maneuver was performed again, generally, after five cardiac cycles, influence of Valsalva maneuver on the cardiac hemodynamics could restore to normal^16^. We quantified shunt severity according to previous criteria^9^: no occurrence of microbubbles appeared in left atrium(negative); grade I, 1 to 10 microbubbles in left atrium; grade II, 10 to 30 microbubbles in left atrium; grade III, more than 30 microbubbles in left atrium or left atrium nearly filled with microbubbles or opacity. All operations were in store for further analysis retrospectively by two experienced sonographers blinded to the result, both at rest or during Valsalva maneuver. Finally, only 55 patients were confirmed PFO by TTE with the micro-bubbles test.

### Statistical Analysis

Categorical and continuous variables were expressed as percentages (%) and mean ± standard deviation, respectively. Statistical analyses were performed using SPSS Version 18.0 (SPSS, Chicago, IL). We used paired t-tests to compare the parameters between the resting and Valsalva maneuver states. A *P* value of <0.05 was considered statistically significant.

## Results

Parameters of left ventricular and right ventricular at rest and during the Valsalva maneuver was shown in Tables 1 and 2. Compared to the resting state, mitral valve diastolic velocity E and A peaks at the strain phase of the Valsalva maneuver significantly decreased (P < 0.05, E peak decreased18.0%, A peak decreased 10.6%), left ventricular end diastolic volume and area decreased significantly (P < 0.05, LVEDV decreased 37.6%, LVEDA decreased 21.7%), while E/A ratio of mitral valve, tricuspid valve systolic velocity E and A peaks and E/A ratio remained unchanged (*P* > 0.05). Results of TTE with the microbubbles test at rest and during the Valsalva maneuver were demonstrated in Table 3. Among the 65 patients, the percentage of positive tests was 58.5% (38 patients) at rest. When the VM was performed, the test result was positive in 55 patients (84.6%). With respect to bubble grade, Valsalva maneuver yielded more positive results than at rest during Grade I shunt and Grade II shunt.

## Discussion

Our results indicated that compared to the resting state, mitral valve diastolic velocity E peak and A peak at the strain phase of the Valsalva maneuver significantly decreased (*P* < 0.05), left ventricular end diastolic volume (LVEDV) and area (LVEDA) decreased significantly (*P* < 0.05), while E/A ratio of mitral valve, tricuspid valve systolic velocity, E and A peak and E/A ratio remained unchanged (*P* > 0.05). The results also indicated that the Valsalva maneuver significantly increased the number of micro-bubbles shunting.

The Valsalva maneuver has been used in clinical practice in patients by changing intrathoracic pressure and causing cardiovascular hemodynamic changes to improve diagnosis accuracy. However, a consensus still has not been reached about a possible mechanism. Some authors^10^,^12^,^17^ held that during the strain phase of the Valsalva maneuver, venous return of vena systemic and right heart system were previously decreased, and after 4–5 cardiac cycles, reduced right ventricular blood volume passed through pulmonary vessels and rushed into the left ventricle, causing a reduction of left ventricular blood volume. Left and right ventricular stroke volumes of normal subjects were nearlly the same, because they could maintain a relative balance owing to series connection of systemic circulation and pulmonary circulation. After 4–5 cardiac cycles of the Valsalva maneuver, left and right ventricles tended to return to normal. While other authors^13^ considered that the increased pressure of the thoracic cavity directly acted on the pericardium and the ventricular free wall during the strain phase of the Valsalva maneuver, the intrathoracic pressure direction was nearly the same direction of ventricular contraction at systolic, but opposed the ventricular diastolic direction. Therefore, we considered that increased intrathoracic positive pressure during the Valsalva maneuver was beneficial to ventricular contraction, while the ventricular filling was limited. All the above changes resulted in mitral valve diastolic velocity E peak, A peak decrease, left ventricular end diastolic volume and area decrease. According to Laplace’s Law, the increased intrathoracic pressure resulted in transversal pressure of cardiac and return venous system, and caused resistance of cardiac and returned venous system increase, thus, left ventricular inflow blood flow spectrum of E peak, A peak, left ventricular end diastolic volume, and area reduced accordingly.

**Figure 5:**
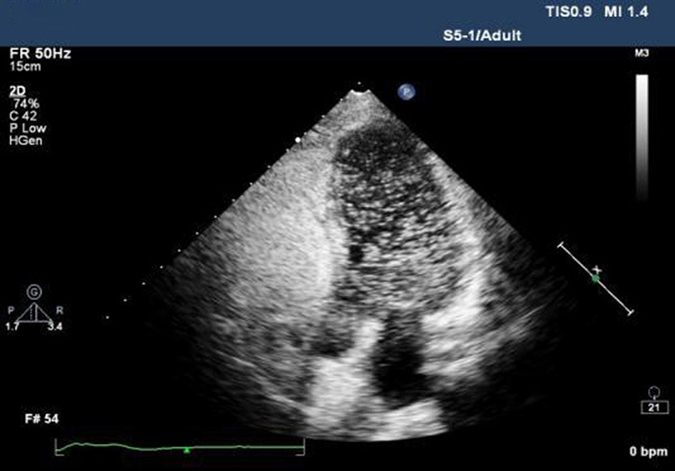
Patent foramen instantaneous opened after the release phase of the Valsalva maneuver.

Among all 65 patients included, the percentage of positive tests was 58.5% (38/65) at rest. When the Valsalva maneuver was performed, the test result was positive in 55 patients (84.6%). The results were consistent with previous studies^9^,^18^. At rest, the left atrial pressure exceeded right atrial pressure, and the left to right shunt via patent foramen ovale generally occurred. Right to left shunt did not occur at the strain phase of the Valsalva maneuver, but after the release phase of the Valsalva maneuver, when the patent foramen instantaneous opened^19^ (Fig. 5). The potential mechanisms were explained as follows: At the strain phase of the standard Valsalva maneuver, intrathoracic pressure was maintained at 40 mmHg. The heart was confined to the chest cavity while systemic circulation vein was located outside the chest cavity. Since the increased intrathoracic pressure resulted in higher blood return resistance, when venous returned to right shunt via superior and inferior vena cava, right ventricular filling decreased. This caused the systemic circulation vein volume to increase temporarily, and systemic circulation vein pressure increased. Besides, at the strain phase of the Valsalva maneuver, intrathoracic positive pressure was beneficial to left ventricular contraction, while left ventricular filling was limited. The left and right heart systems were connected in series. When the reduced right heart blood volume passed through the pulmonary circulation, pulmonary venous blood volume also decreased. Pulmonary venous blood volume returned to left ventricular further decreased, and left atrial filling pressure further reduced. Here, the right atrial pressure may be slightly higher than or equal to the left atrial pressure, when right heart contrast echocardiography was carried out, some scattered visible microbubbles passed through the foramen ovale, namely the right to left shunt. After the release phase of the Valsalva maneuver, intrathoracic pressure reduced instantly, blood volume returned to the right atrium from superior and inferior vena cava which increased instantaneously, and the right atrial pressure also corresponding increased transiently. Blood volume returned to the left atrium, which mainly came from the pulmonary vein after the release phase of Valsalva maneuver. Blood return resistance was removed, residual blood volume in the lung returned to the left atrium via pulmonary vein, and less blood volume returned to the right atrium via the systemic circulation vein^13^. Thus, right atrial pressure was significantly higher than left atrial pressure, patent foramen ovale opened, microbubbles passed through the open channel, and the left atrium was filled with a large number of microbubbles.

However, our work also had limitations. First, the major problem was the small number of sample size with only 55 cases of patent foramen ovale confirmed by TTE with the micro-bubbles test. Second, there is no apparent power calculation in our work and given the drop-out of non-PFO patients present, it is likely that type I statistical errors could be present. Besides, our patients did not undergo transesophageal echocardiography (TEE) to confirm the presence of patent foramen ovale.

In conclusion, our results indicated that patients with patent foramen ovale hemodynamic changes mainly occurred in the left ventricle when the Valsalva maneuver was performed, mitral valve diastolic velocity E peak and A peak at the strain phase of Valsalva maneuver significantly decreased, left ventricular end diastolic volume (LVEDV) and area (LVEDA) decreased significantly, while E/A ratio of mitral valve, tricuspid valve systolic velocity E peak, A peak and E/A ratio remained unchanged. The results also indicated that the Valsalva maneuver significantly increased the number of micro-bubbles shunting. The Valsalva maneuver increased the pressure of the chest, and pulmonary venous return was impeded, which resulted in left ventricular filling, and E and A peaks decreased. The pressure of left ventricle and atrium was lower than that of the right side, which resulted in right-to- left shunt (RLS) through PFO.

## Additional Information

**How to cite this article**: Zhao, E. *et al*. Influence of the Valsalva maneuver on the cardiac hemodynamics and right to left shunt in patients with patent foramen ovale. *Sci. Rep.* **7**, 44280; doi: 10.1038/srep44280 (2017).

**Publisher's note:** Springer Nature remains neutral with regard to jurisdictional claims in published maps and institutional affiliations.

## Figures and Tables

**Table 1:** Parameters of the left ventricle and P values at rest and during the Valsalva maneuver.

	E(cm/s)	A(cm/s)	E/A	LVEDV(ml)	LVEDA(cm^2^)
At rest	78.2 ± 18.2	58.4 ± 14.90	1.5 ± 0.60	69.0 ± 14.8	24.40 ± 3.8
Valsalva maneuver	64.1 ± 16.2	52.2 ± 16.0	1.30 ± 0.50	43.0 ± 16.3	19.1 ± 5.1
P	<0.01	<0.05	0.081	<0.01	<0.01

LVEDV = left ventricular end-diastolic volume, LVEDA = left ventricular end-diastolic area.

**Table 2:** Parameters of the right atrium and P values at rest and during the Valsalva maneuver.

	E(cm/s)	A(cm/s)	E/A	RAEA(cm^2^)
At rest	53.4 ± 14.1	42.0 ± 10.7	1.30 ± 0.1	12.8 ± 3.8
Valsalva maneuver	53.5 ± 16.3	42.0 ± 16.1	1.4 ± 0.1	8.2 ± 3.9
P	0.863	0.986	0.479	<0.01

RAEA = right atrium area.

**Table 3:** Results of TTE with the microbubbles test at rest and during the Valsalva maneuver.

	at rest	Valsalva maneuver
Negative	27 (41.5%)	10 (15.4%)
Positive	38 (58.5%)	55 (84.6%)
Grade I	23	8
Grade II	8	13
Grade III	7	34

TTE = Transthoracic echocardiography.

## References

[b1] Hagen P. T., Scholz D. G. & Edwards W. D. Incidence and size of patent foramen ovale during the first 10 decades of life: an autopsy study of 965 normal hearts. Mayo Clinic proceedings 59, 17–20 (1984). 6694427 10.1016/s0025-6196(12)60336-x

[b2] Ghosh A. K. & Jain A. Diagnosis and management of patent foramen ovale. British journal of hospital medicine (London, England: 2005) 76, C98–102, doi: 10.12968/hmed.2015.76.7.C98 (2015). 26140572

[b3] Buchholz S., Shakil A., Figtree G. A., Hansen P. S. & Bhindi R. Diagnosis and management of patent foramen ovale. Postgraduate medical journal 88, 217–225, doi: 10.1136/postgradmedj-2011-130368 (2012). 22282739

[b4] Rigatelli G. & Rigatelli A. Closing patent foramen ovale in cryptogenic stroke: The underscored importance of other interatrial shunt variants. World journal of cardiology 7, 326–330, doi: 10.4330/wjc.v7.i6.326 (2015). 26131337 PMC4478567

[b5] Wozniak L., Mielczarek M. & Sabiniewicz R. Paradoxical brain embolism in a young man: is it only a patent foramen ovale? Neurologia i neurochirurgia polska 49, 61–64, doi: 10.1016/j.pjnns.2014.12.003 (2015). 25666776

[b6] Mojadidi M. K., Gevorgyan R., Noureddin N. & Tobis J. M. The effect of patent foramen ovale closure in patients with platypnea-orthodeoxia syndrome. Catheterization and cardiovascular interventions: official journal of the Society for Cardiac Angiography & Interventions 86, 701–707, doi: 10.1002/ccd.25953 (2015). 26063336

[b7] Asrress K. N., Marciniak M., Marciniak A., Rajani R. & Clapp B. Patent foramen ovale: the current state of play. Heart (British Cardiac Society) 101, 1916–1925, doi: 10.1136/heartjnl-2015-307639 (2015). 26487104

[b8] Merante A. . Transient cerebral ischemia in an elderly patient with patent foramen ovale and atrial septal aneurysm. Clinical interventions in aging 10, 1445–1449, doi: 10.2147/cia.s80190 (2015). 26379429 PMC4567244

[b9] Zhao E. . A Comparison of Transthroracic Echocardiograpy and Transcranial Doppler With Contrast Agent for Detection of Patent Foramen Ovale With or Without the Valsalva Maneuver. Medicine 94, e1937, doi: 10.1097/md.0000000000001937 (2015). 26512622 PMC4985435

[b10] Nishimura R. A. & Tajik A. J. The Valsalva maneuver-3 centuries later. Mayo Clinic proceedings 79, 577–578, doi: 10.4065/79.4.577 (2004). 15065629

[b11] Gentile M., De Vito A., Azzini C., Tamborino C. & Casetta I. Adding blood to agitated saline significantly improves detection of right-to-left shunt by contrast-transcranial color-coded duplex sonography. Ultrasound in medicine & biology 40, 2637–2641, doi: 10.1016/j.ultrasmedbio.2014.06.017 (2014). 25220269

[b12] Zhang R., Crandall C. G. & Levine B. D. Cerebral hemodynamics during the Valsalva maneuver: insights from ganglionic blockade. Stroke; a journal of cerebral circulation 35, 843–847, doi: 10.1161/01.str.0000120309.84666.ae (2004). 14976327

[b13] Wang Z. . Simultaneous beat-by-beat investigation of the effects of the Valsalva maneuver on left and right ventricular filling and the possible mechanism. PloS one 8, e53917, doi: 10.1371/journal.pone.0053917 (2013). 23342040 PMC3544743

[b14] Nagueh S. F. . Recommendations for the evaluation of left ventricular diastolic function by echocardiography. European journal of echocardiography: the journal of the Working Group on Echocardiography of the European Society of Cardiology 10, 165–193, doi: 10.1093/ejechocard/jep007 (2009). 19270053

[b15] Lang R. M. . Recommendations for chamber quantification: a report from the American Society of Echocardiography’s Guidelines and Standards Committee and the Chamber Quantification Writing Group, developed in conjunction with the European Association of Echocardiography, a branch of the European Society of Cardiology. Journal of the American Society of Echocardiography: official publication of the American Society of Echocardiography 18, 1440–1463, doi: 10.1016/j.echo.2005.10.005 (2005). 16376782

[b16] Porth C. J., Bamrah V. S., Tristani F. E. & Smith J. J. The Valsalva maneuver: mechanisms and clinical implications. Heart & lung: the journal of critical care 13, 507–518 (1984). 6565684

[b17] Mortara A. . Arterial baroreflex modulation of heart rate in chronic heart failure: clinical and hemodynamic correlates and prognostic implications. Circulation 96, 3450–3458 (1997). 9396441 10.1161/01.cir.96.10.3450

[b18] Clarke N. R., Timperley J., Kelion A. D. & Banning A. P. Transthoracic echocardiography using second harmonic imaging with Valsalva manoeuvre for the detection of right to left shunts. European journal of echocardiography: the journal of the Working Group on Echocardiography of the European Society of Cardiology 5, 176–181, doi: 10.1016/s1525-2167(03)00076-3 (2004). 15147659

[b19] Paliwal P. R. & Sharma V. K. Valsalva maneuver in detection of right-to-left shunt by transcranial Doppler. Arquivos de neuro-psiquiatria 68, 979 (2010). 21243270 10.1590/s0004-282x2010000600034

